# Transgenic tobacco plant overexpressing ginkgo dihydroflavonol 4-reductase gene *GbDFR6* exhibits multiple developmental defects

**DOI:** 10.3389/fpls.2022.1066736

**Published:** 2022-12-14

**Authors:** Jun Ni, Ning Zhang, Yang Zhan, Kexin Ding, Peng Qi, Xuejun Wang, Wona Ding, Maojun Xu

**Affiliations:** ^1^ Key Laboratory of Hangzhou City for Quality and Safety of Agricultural Products, College of Life and Environmental Sciences, Hangzhou Normal University, Hangzhou, China; ^2^ Zhejiang Provincial Key Laboratory for Genetic Improvement and Quality Control of Medicinal Plants, Hangzhou Normal University, Hangzhou, China; ^3^ College of Science and Technology, Ningbo University, Ningbo, China

**Keywords:** anthocyanin, auxin, dihydroflavonol 4-reductase, flavonoid, flowering, ginkgo, tobacco

## Abstract

Dihydroflavonol Q 4-reductase (DFR), a key enzyme in the flavonoid biosynthetic pathway in plants, significantly influences plant survival. However, the roles of DFR in the regulation of plant development are largely unknown. In the present study, phenotypes of transgenic tobacco plants overexpressing the *Ginkgo biloba DFR* gene, *GbDFR6*, were investigated. Transgenic tobacco seedlings exhibited relatively low fresh weights, long primary roots, decreased lateral root numbers, and impaired root gravitropic responses when compared to wild-type tobacco plants. Adult transgenic tobacco plants exhibited a considerably high percentage of wrinkled leaves when compared to the wild-type tobacco plants. In addition to the auxin-related phenotypic changes, transgenic tobacco plants exhibited delayed flowering phenotypes under short-day conditions. Gene expression analysis revealed that the delayed flowering in transgenic tobacco plants was caused by the low expression levels of *NtFT4*. Finally, variations in anthocyanin and flavonoid contents in transgenic tobacco plants were evaluated. The results revealed that the levels of most anthocyanins identified in transgenic tobacco leaves increased. Specifically, cyanidin-3,5-*O*-diglucoside content increased by 9.8-fold in transgenic tobacco plants when compared to the wild-type tobacco plants. Pelargonidin-3-*O*-(coumaryl)-glucoside was only detected in transgenic tobacco plants. Regarding flavonoid compounds, one flavonoid compound (epicatechin gallate) was upregulated, whereas seven flavonoid compounds (Tamarixetin-3-*O*-rutinoside; Sexangularetin-3-*O*-glucoside-7-*O*-rhamnoside; Kaempferol-3-*O*-neohesperidoside; Engeletin; 2’-Hydoxy,5-methoxyGenistein-*O*-rhamnosyl-glucoside; Diosmetin; Hispidulin) were downregulated in both transgenic tobacco leaves and roots. The results indicate novel and multiple roles of *GbDFR6* in ginkgo and provide a valuable method to produce a late flowering tobacco variety in tobacco industry.

## Introduction

Dihydroflavonol 4-reductase (DFR, EC1.1.1.219), a key enzyme in the regulation of the carbon flux direction in the flavonoid biosynthetic pathway, catalyzes the reduction of the 4-keto group of dihydroflavonol to the corresponding leucoanthocyanidin, which is the first committed reaction leading to anthocyanin production (Saito et al., 2013). Anthocyanins perform multiple functional roles in plant–environment interactions, which protect plants from biotic and abiotic stresses (Landi et al., 2015). Overexpression of *DFR* genes leads to an increase in anthocyanin content, and the altered phenotypes of transgenic plants reflect the physiological roles of anthocyanins in plants (Polashock et al., 2002; Kumar et al., 2013; Shin et al., 2016; Ni et al., 2020).

The phytohormone auxin is critical for plant growth and it influences many developmental processes (Woodward and Bartel, 2005). Auxin is produced at its synthesis sites and then distributed throughout the whole plant by a variety of auxin transporters to regulate most plant growth and developmental processes (Swarup and Bennett, 2003; Rozov et al., 2013). The inhibition of auxin transport leads to several defects, including delayed gravitropic responses (Rashotte et al., 2000), and altered root and leaf development (Friml, 2003; Overvoorde et al., 2010). The regulation of auxin transport has been investigated using both artificial and naturally occurring auxin transport inhibitors, with flavonoids being one of the naturally occurring auxin transport inhibitors (Peer and Murphy, 2007). Although one flavonoid compound (kaempferol 3-*O*-rhamnoside-7-*O*-rhamnoside) has been demonstrated to act as an endogenous polar auxin transport inhibitor in *Arabidopsis* (Yin et al., 2014), our knowledge of auxin transport regulation by flavonoids remains limited.

Flowering time is essential for plant reproduction and is an important trait in agriculture. Numerous studies have revealed that FLOWERING LOCUS T (FT)/TERMINAL FLOWER 1 (TFL1) protein family is a key factor influencing the regulation of flowering time (Wickland and Hanzawa, 2015). Many environmental cues, such as photoperiod, vernalization, and high ambient temperatures, as well as endogenous cues, such as plant age, the phytohormone gibberellin, and carbohydrate status, affect plant flowering time through the central FT/TFL1 regulatory hubs (Ponnu et al., 2011; Andres and Coupland, 2012; Capovilla et al., 2015; Kinoshita and Richter, 2020). However, despite the numerous factors regulating *FT/TFL1* gene expressions, the role of flavonoids in the regulation of flowering time remains largely unknown.

*Ginkgo biloba* is a perennial gymnosperm, which is one of the most popular medicinal plants worldwide. Flavonoids are the main bioactive constituents among the numerous secondary metabolites in ginkgo leaf extracts that are associated with pharmacological activities (Singh et al., 2008). The flavonoid biosynthesis pathway and the various environmental factors affecting the pathway in ginkgo leaves have been investigated, including salt (Ni et al., 2017), circadian rhythms (Ni et al., 2018a) and salicylic acid (Ni et al., 2018b). Further research reported differently changed contents of flavonoids in ginkgo when facing specific environmental stresses (Ni et al., 2020). Additionally, as one of the world’s most widely used ornamental street trees, ginkgo has become a popular horticultural novelty. With its distinctive branching and fan-shaped leaves, ginkgo is featured prominently in all types of art (Crane, 2019). However, despite the distinctive appearance and popularity of ginkgo, the molecular mechanisms regulating its development remain poorly understood.

Up to now, a total of six *DFR* genes have been isolated in ginkgo. The functions of these DFRs in the anthocyanin biosynthesis have been confirmed, and their roles in environmental stress responses have been explored (Hua et al., 2013; Ni et al., 2020). Here, we further investigated the phenotypic changes in transgenic tobacco (*Nicotiana tabacum*) plants overexpressing *GbDFR6*. Pleiotropic defects in root and leaf development indicated impaired auxin transport in transgenic tobacco plants. Delayed flowering in transgenic tobacco plants signified the involvement of anthocyanins (or flavonoids) in the regulation of flowering time in plants. The results of the present study indicate the novel and multiple roles of *GbDFR6* in ginkgo and provide a valuable method to produce a late flowering tobacco variety in tobacco industry.

## Materials and methods

### Plant materials and growth conditions

The genetic information of transgenic tobacco plants (*N. tabacum* cv. K326) overexpressing *GbDFR6* has been described in a previous study (Ni et al., 2020). Tobacco seeds were sterilized with 75% ethanol and planted in plates containing Murashige and Skoog salts (Gibco, Grand Island, NY, USA) and 1.5% (w/v) agar (Becton Dickinson Vacutainer Systems, Rutherford, NJ, USA). Seedlings were grown vertically in growth chambers under short-day (SD) conditions (8 h of light and 16 h of darkness) at a constant temperature of 23°C. To obtain adult plants, two-week-old tobacco seedlings were transferred into pots containing nutrient-rich soils (HAWITA, Vogelsberg, Germany). The growth conditions of adult plants were the same as those for tobacco seedlings. To perform the root gravitropic response assay, ten wild-type and ten transgenic tobacco seedlings were grown horizontally in plates for one month. Gravitropic stimulation was achieved by rotating the plates through 90°. The bending angles of root tips were measured every 24 h. The experiment was repeated for three times. To distinguish between moderately and severely wrinkled leaves, one moderately wrinkled leaf was considered to be equal to half of a wrinkled leaf.

### Auxin measurement of tobacco seedlings

Leaves and roots of one-month-old tobacco seedlings were harvested separately for auxin measurements. Leaf and root tissues were weighted immediately after harvest. Then, these tissues were homogenized in PBS solution (pH 7.4) by hand and centrifuged at the seed of 1000 ×*g* for 20 min. The supernatant was used in the auxin measurements. Measurements of auxin levels were carried out following the instructions of the Auxin ELISA kit (Jingmei Biotechnology, Jiangsu, China).

### Characterization of flowering in tobacco plants

To compare the expression levels of *FT* genes between transgenic and wild-type tobacco plants, plants were grown under either SD or long-day (LD) conditions at a constant temperature of 23°C. Tobacco leaves were harvested just before bolting (approximately 90 days after germination for SD and approximately 60 days after germination for LD). No marked phenotypic variations were observed between transgenic and wild-type tobacco plants. After RNA extraction and first-strand complementary DNA synthesis, quantitative reverse transcription polymerase chain reaction (qRT-PCR) was performed to examine the expression levels of four *FT-*like genes (*NtFT1*, *NtFT2*, *NtFT3*, and *NtFT4*) using the primers listed in **Table S1**. The relative expression level of each gene was determined by normalizing to the expression level of the *NtEF1α* gene in the same sample using the method of REST-MCS as described (Harig et al., 2012). Flowering time was determined when the first flower opened.

### Extraction and quantification of anthocyanin and flavonoid contents in tobacco leaves

The processes of extraction, separation and quantification were modified from the previous research (Fraga et al., 2010; Chen et al., 2013).

The freeze-dried tobacco leaf samples were crushed using a mixer mill (MM 400, Retsch, GmbH, Haan, Germany) with a zirconia bead for 1.5 min at a frequency of 30 Hz. Subsequently, 50 mg powder was weighed and extracted with 0.5 mL 70% aqueous methanol. The extract was vortexed for 5 min, subjected to ultrasound (50W and 0°C) for 5 min, and centrifuged for 3 min at 12,000 ×*g* and 4°C. The residue was extracted twice by repeating the previously mentioned steps. The supernatants were collected and filtered using 0.22-μm filters (ANPEL Laboratory Technologies Inc., Shanghai, China) for subsequent liquid chromatography-tandem mass spectrometry (LC-MS/MS) analysis.

The sample extracts were analyzed using an ultra-performance liquid chromatography-electrospray ionization-tandem mass spectrometry (UPLC-ESI-MS/MS) system (UPLC, ExionLC™ AD, https://sciex.com.cn/; MS, Applied Biosystems 6500 Triple Quadrupole, https://sciex.com.cn/). The system was equipped with a UPLC column (Waters Acquity BEH C18, 1.7 µm, 2.1 mm × 100 mm). The solvent system comprised 0.1% formic acid in water and 0.1% formic acid in methanol. The gradient program was as follows: 95:5 v/v at 0 min, 50:50 v/v at 6 min, 5:95 v/v at 12 min, hold for 2 min, 95:5 v/v at 14 min; hold for 2 min. The flow rate was set at 0.35 mL/min; temperature was maintained at 40°C, and injection volume was 2 μL. The detection wavelength was 360 nm. The effluent was alternatively connected to an ESI-triple quadrupole-linear ion trap (QTRAP)-MS.

The linear ion trap and triple quadrupole scans were acquired using a QTRAP mass spectrometer (API 6500 QTRAP UPLC/MS/MS System) equipped with an ESI turbo ion spray interface, which was operated in the positive ion mode and controlled by Analyst 1.6.3 software (AB SCIEX, Foster City, CA, USA). The ESI source operation parameters were as follows: ion source was turbo ion spray; source temperature was 550°C; ion spray voltage of 5500 V (positive ion mode); curtain gas was set at 35 psi. D*eclustering potential* (DP) and collision energy (CE) for individual multiple reaction monitoring (MRM) transitions were performed by optimizing DP and CE. A specific set of MRM transitions was monitored for each period based on the metabolites eluted within the period.

## Results

### Transgenic tobacco seedlings overexpressing *GbDFR6* exhibit pleiotropic root defects

**Figure 1:**
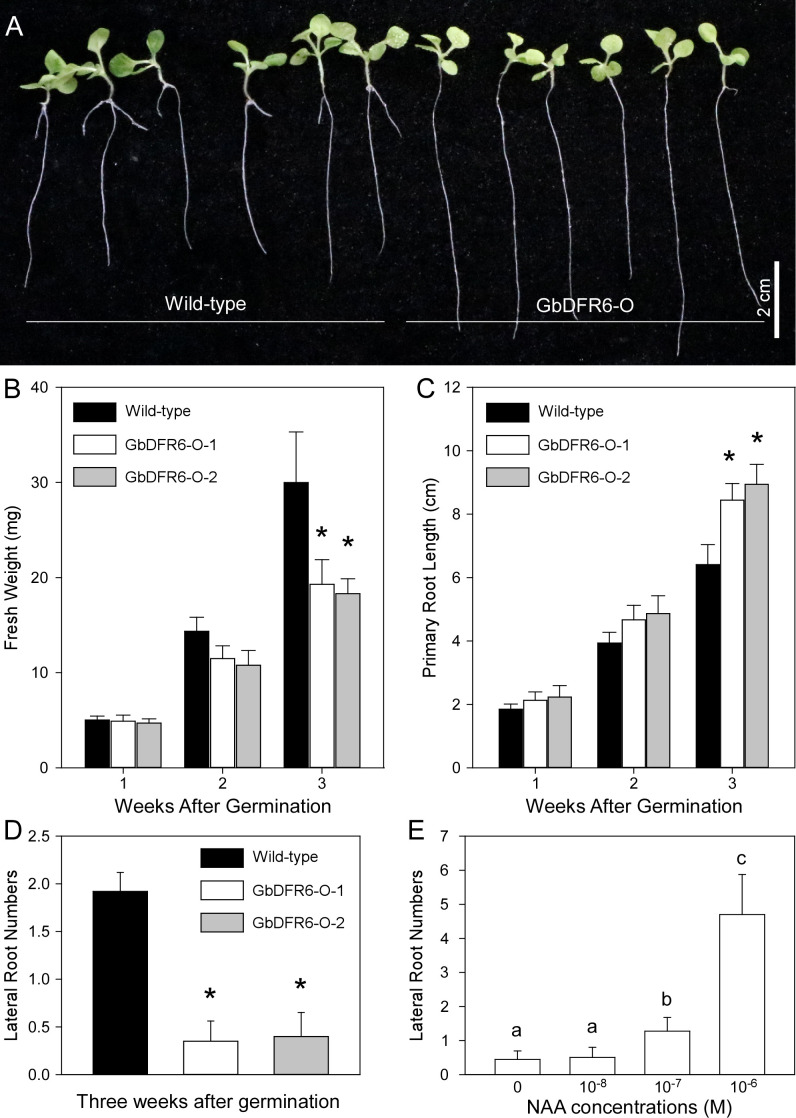
A comparison of growth parameters between wild-type and transgenic tobacco seedlings. **(A)** Three-week-old wild-type and transgenic tobacco seedlings. Bar = 2 cm. **(B)** A Comparison of fresh weights between wild-type and transgenic tobacco seedlings during the first three weeks after germination. **(C)** A comparison of primary root lengths between wild-type and transgenic tobacco seedlings during the first three weeks after germination. **(D)** A comparison of lateral root numbers between wild-type and transgenic tobacco seedlings three weeks after germination (data are presented as means ± SD; n = 20; * indicates significant differences, *P* < 0.01; Student’s *t*-test). SD, standard deviation. **(E)** The induction of lateral root of transgenic tobacco seedlings by exogenous auxin treatment (n = 20; Different letters indicate significant differences, *P* < 0.01; Student’s *t*-test).

To investigate the functions of GbDFR6 products in plant development, phenotypes of wild-type and transgenic tobacco plants overexpressing the *GbDFR6* gene were compared. The total fresh weights, primary root lengths, and lateral root numbers of wild-type and transgenic tobacco seedlings were measured and compared. Overall, the transgenic tobacco seedlings developed more slowly than wild-type tobacco seedlings (**Figure 1A**). Moreover, the differences in weights increased during the first three weeks after germination (WAGs) (**Figure 1B**). In contrast, the primary roots of transgenic tobacco seedlings were longer than those of the wild-type tobacco seedlings, and the differences in root lengths increased during the first three WAGs (**Figures 1A, C**). In addition, transgenic tobacco seedlings had fewer lateral roots than the wild-type tobacco seedlings (**Figures 1A, D**). Lateral root growth in transgenic tobacco seedlings was induced by exogenous auxin treatment (**Figures 1E**, **S1**).

**Figure 2:**
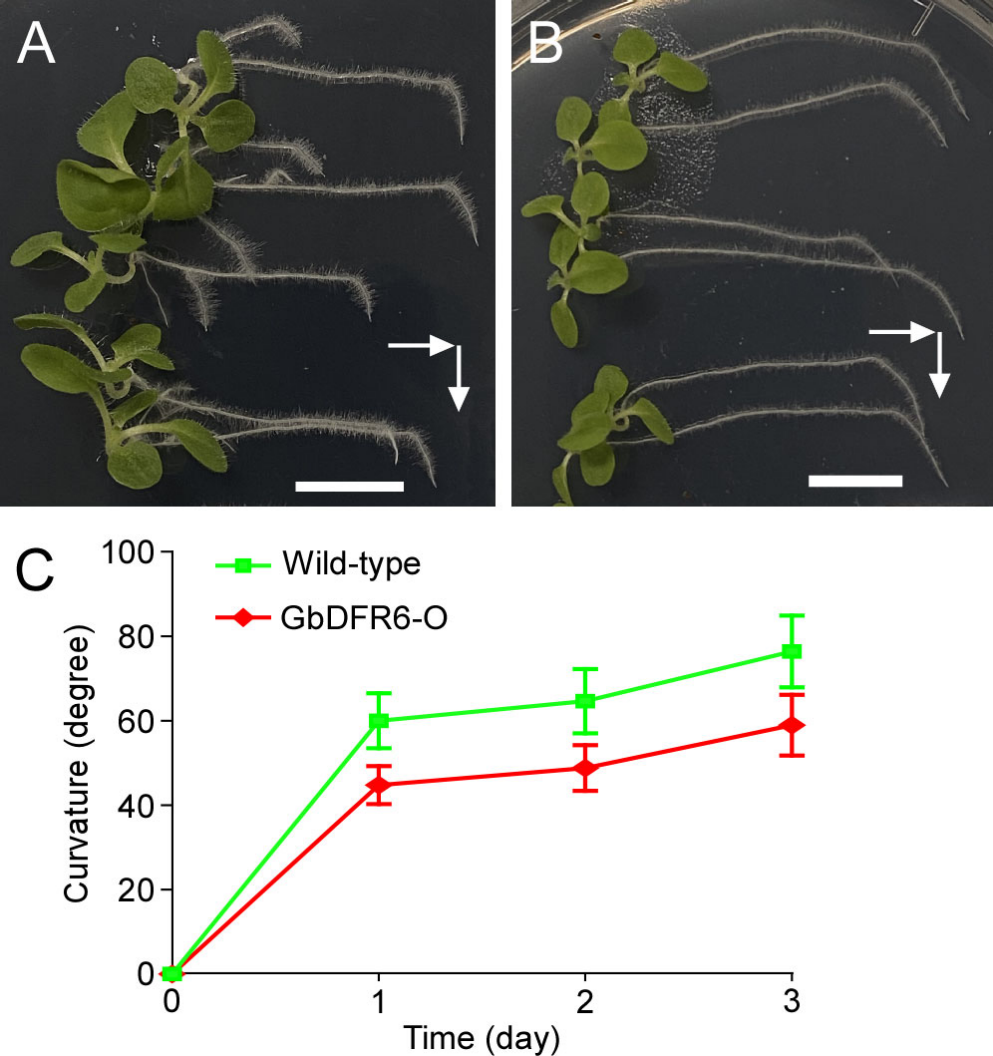
Gravitropic responses of wild-type and transgenic tobacco roots. One-month-old wild-type **(A)** and transgenic **(B)** tobacco seedlings were laid down from an upright to horizontal position. White arrows indicate gravitational directions. Bars = 1 cm. **(C)** The curvature of the primary roots at different time points. The green line represents wild-type tobacco plants, whereas the red line represents transgenic tobacco plants. Means and SDs are shown (n = 10).

Root gravitropism in transgenic tobacco plants was also affected. Gravistimulation led to the downward bending of the primary roots when one-month-old tobacco seedlings were placed horizontally in the growth chambers. The primary roots of wild-type tobacco seedlings bent rapidly after gravistimulation, resulting in a sharp bend (**Figure 2A**). In contrast, the primary roots of transgenic tobacco seedlings bent gradually after gravistimulation, resulting in a smooth curved bend (**Figure 2B**). In addition, the bending of the primary roots was significantly delayed in transgenic tobacco plants (**Figure 2C**). Therefore, the regulation of root gravitropism was impaired in transgenic tobacco seedlings.

Next, the overall auxin contents in leaves and roots were measured and compared. No significant differences in auxin contents were observed between wild-type and transgenic seedlings either in leaves or in roots (**Figure S2**).

### Wrinkling of leaves in transgenic tobacco plants

**Figure 3:**
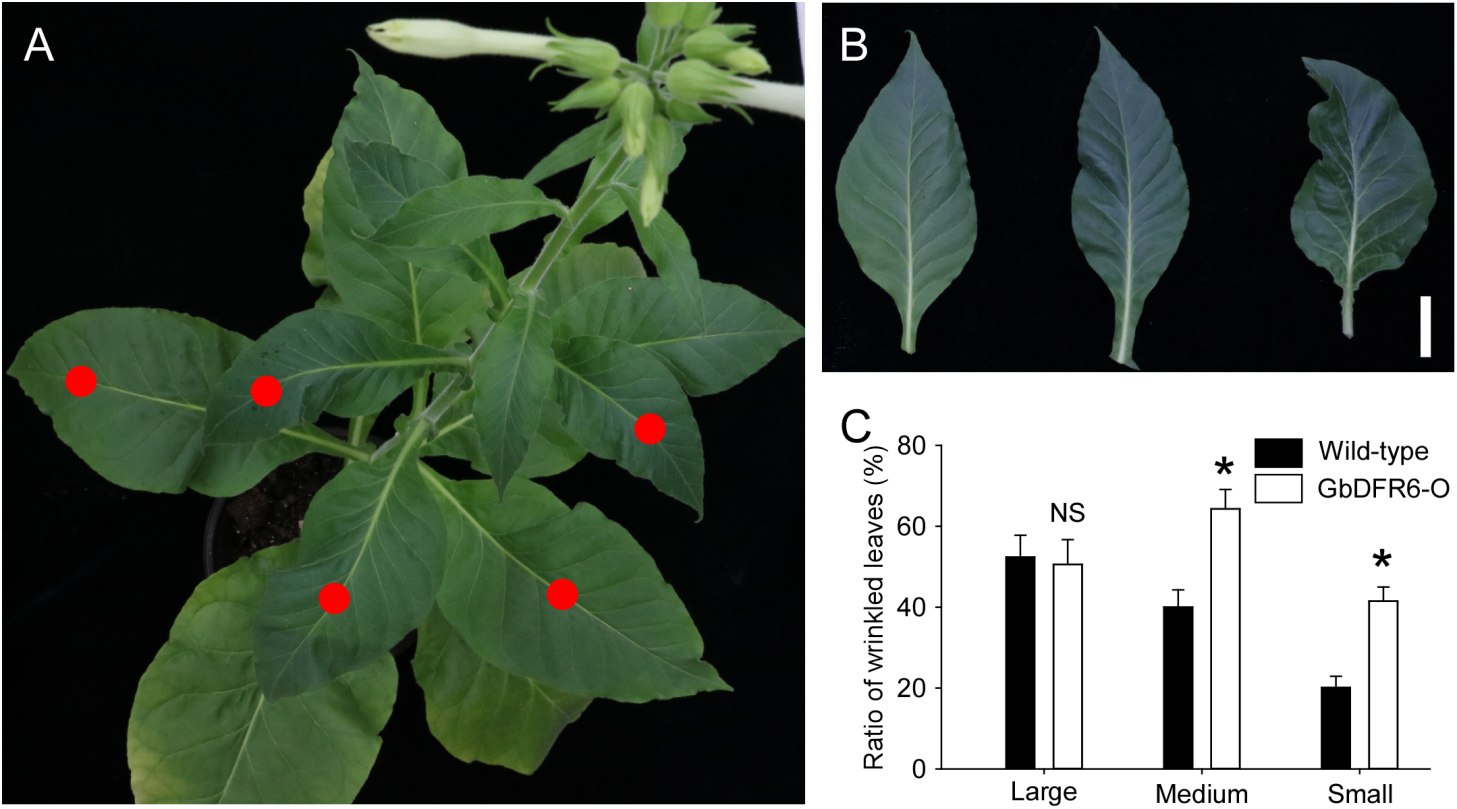
Leaf wrinkling in tobacco plants. **(A)** A typical flowering stage of the wild-type tobacco plant with wrinkled leaves indicated by red dots. **(B)** Varying degrees of tobacco leaf wrinkling. Left, an unfolded leaf; middle, an intermediate wrinkled leaf; right, a severely wrinkled leaf. Bar = 1 cm. **(C)** Comparisons of wrinkled leaf ratios between wild-type and transgenic tobacco plants with small (width < 2 cm), medium (width of 2–7 cm), and large (width > 7 cm) leaves. Data for independent experiments are shown (means ± SD; n = 20; * indicates significant differences, *P* < 0.01; NS, not significant, *P* > 0.05; Student’s *t*-test).

A considerable portion of the leaves in wild-type tobacco plants were wrinkled during the flowering stage (**Figure 3A**). Furthermore, the degree of wrinkling was characterized from intermediate to severe (**Figure 3B**). Approximately 50% of large leaves (width > 7 cm), which occurred at the bottom of the plants, were wrinkled, whereas only 20% of small leaves (width < 2 cm), which occurred at the top of the plants, were wrinkled. Similarly, 50% of large leaves occurring at the bottom of the transgenic tobacco plants were wrinkled when compared to those of the wild-type tobacco plants. However, much higher proportions of medium (width of 2–7 cm) and small leaves in transgenic tobacco plants were wrinkled when compared to those in wild-type tobacco plants (**Figure 3C**). Therefore, the regulation of leaf development, especially medium and small leaves in transgenic tobacco plants, was suppressed.

### Delayed flowering in transgenic tobacco plants under short-day conditions

**Figure 4:**
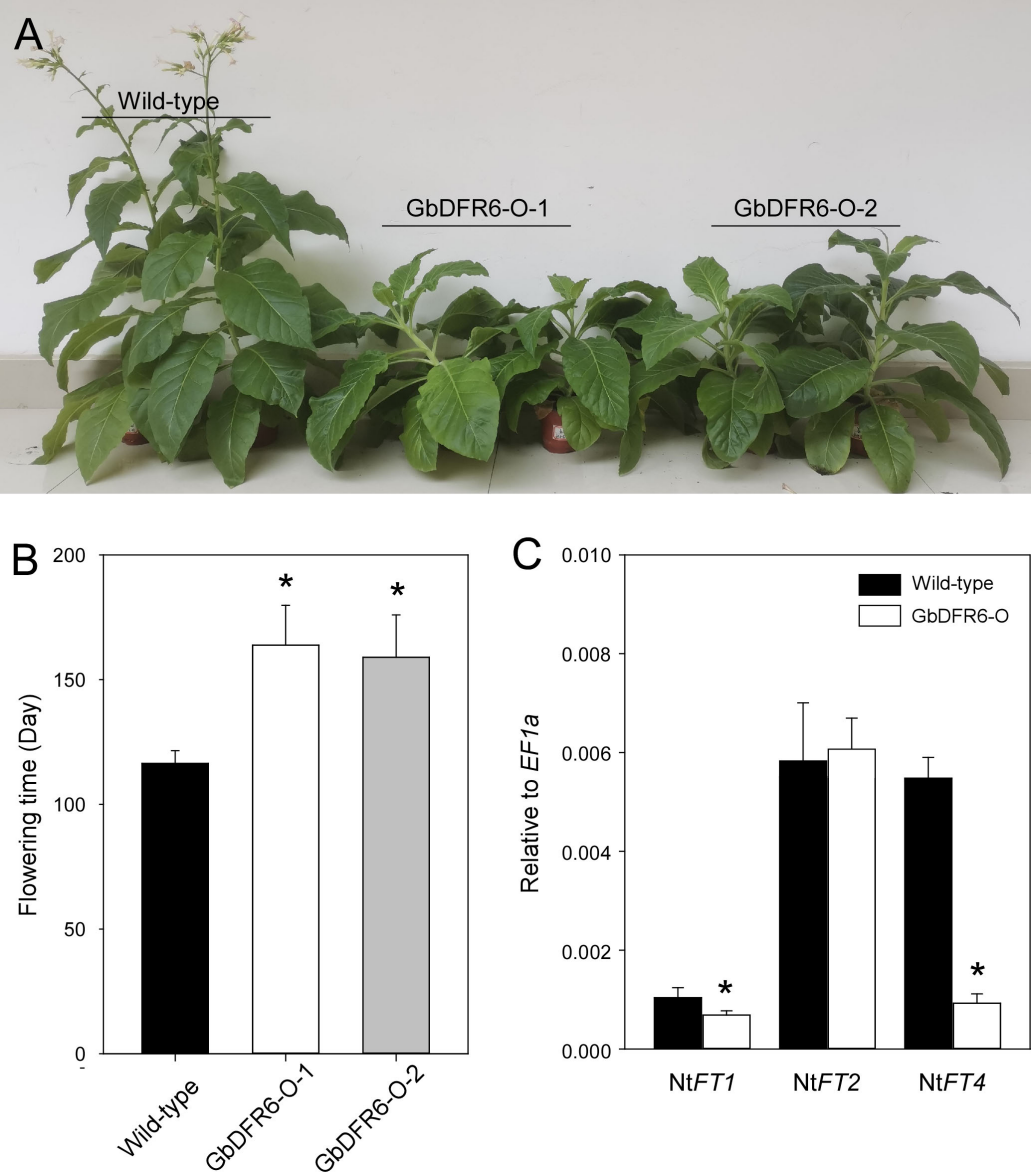
Comparative analysis of flowering time between wild-type and transgenic tobacco plants under short-day conditions. **(A)** Morphological comparison of flowering time between wild-type and transgenic tobacco plants. Two transgenic lines exhibited delayed bolting and flowering times when compared to the wild-type tobacco plants. **(B)** Comparison of flowering days between wild-type and two transgenic tobacco lines (data are presented as means ± SD; n = 10; * indicates significant differences, *P* < 0.01; Student’s *t*-test). **(C)** Expression of *NtFTs* in tobacco plants before bolting as determined by quantitative reverse transcription polymerase chain reaction. The expressions levels of *NtFT1* and *NtFT4* were significantly downregulated in transgenic tobacco plants (data are presented as means ± SD; n = 4; * indicate significant differences, *P* < 0.01; Student’s *t*-test).

In addition to the defects in root and leaf development, flowering was delayed in transgenic tobacco plants overexpressing *GbDFR6* (**Figure 4A**). Wild-type tobacco plants began flowering approximately 120 days after germination under SD conditions, while flowering in transgenic tobacco plants was delayed by approximately 50 days when compared to that in the wild-type tobacco plants (**Figure 4B**). To investigate the mechanism of delayed flowering in transgenic plants, the expression levels of four *FT-*like genes (*NtFT1*, *NtFT2*, *NtFT3*, and *NtFT4*) were analyzed before bolting (Harig et al., 2012). The relative expression levels of *NtFT1* were lower in transgenic tobacco plants than in the wild-type tobacco plants. However, no significant differences were observed in the expression levels of *NtFT2*. The expression levels of *NtFT3* were too low to be detected by qRT-PCR (**Figure 4C**). Notably, the expression levels of *NtFT4*, which is the sole floral inducer (Harig et al., 2012), were considerably downregulated in transgenic tobacco plants (**Figure 4C**). In contrast, no significant differences were observed in flowering time between wild-type and transgenic tobacco plants under LD conditions (**Figures S3A, B**). Gene expression analysis revealed that the overall expression levels of *FT* genes were low and near detection threshold. The expression levels of *NtFT1* and *NtFT3* were too low to be detected, and those of *NtFT2* and *NtFT4* were lower in transgenic tobacco plants than in the wild-type tobacco plants (**Figures S3C, D**).

### Variations in anthocyanin contents in transgenic tobacco leaves

**Figure 5:**
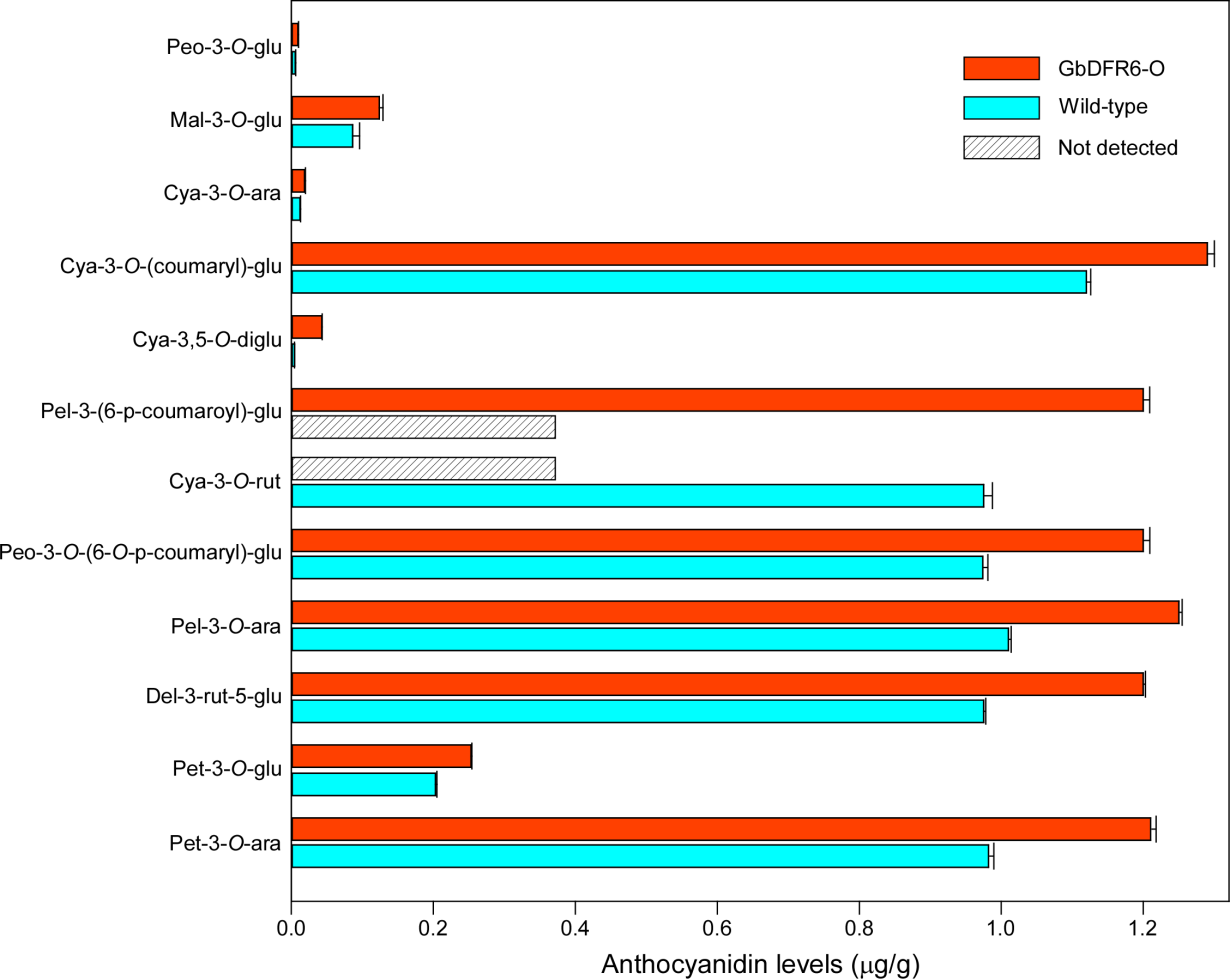
A comparison of various anthocyanin compounds between wild-type and transgenic tobacco leaves. A total of 12 anthocyanin compounds were detected. Pel-3-*O*-(coumaryl)-glu was not detected in wild-type tobacco leaves and cya-3-*O*-rut was not detected in transgenic tobacco leaves. Peo-3-*O*-glu, peonidin-3-*O*-glucoside; Mal-3-*O*-glu, malvidin-3-*O*-glucoside; Cya-3-*O*-ara, Cya-3-*O*-(coumaryl)-glu, Cya-3,5-*O*-diglu, cyanidin-3,5-*O*-diglucoside; Pel-3-(6-p-coumaroyl)-glu, pelargonidin-3-*O*-(coumaryl)-glucoside; Cya-3-*O*-rut, cyaniding-3-*O*-rutinoside; Peo-3-*O*-(6-*O*-p-coumaryl)-glu, peonidin-3-*O*-(6-*O*-p-coumaryl)-glucoside; Pel-3-*O*-ara, pelargonidin-3-*O*-arabinoside; Del-3-rut-5-glu, delphinidin-3-rutinoside-5-glucoside; Pet-3-*O*-glu, petunidin-3-*O*-glucoside; Pet-3-*O*-ara, petunidin-3-*O*-arabinoside. Three biological repetitions were carried out during the experiment.

According to a previous study, the total anthocyanin content in transgenic tobacco plants increased when compared to that in wild-type tobacco plants (Ni et al., 2020). However, the various anthocyanin components were not investigated. To further explore the variations in the different anthocyanin components caused by the overexpression of *GbDFR6* in transgenic tobacco plants, an UPLC-ESI-MS/MS system was used to compare individual anthocyanin contents between wild-type and transgenic tobacco leaves. A total of 12 anthocyanin components were detected in wild-type and transgenic tobacco leaves. In addition, cyanidin-3-*O*-rutinoside and pelargonidin-3-*O*-(coumaryl)-glucoside were specifically detected in wild-type and transgenic tobacco leaves, respectively. The contents of all anthocyanin components identified in transgenic tobacco leaves increased, with cyanidin-3,5-*O*-diglucoside exhibiting a 9.8-fold change (**Figures 5**, **S4**).

### Flavonoid contents in transgenic and wild-type tobacco leaves and roots

**Figure 6:**
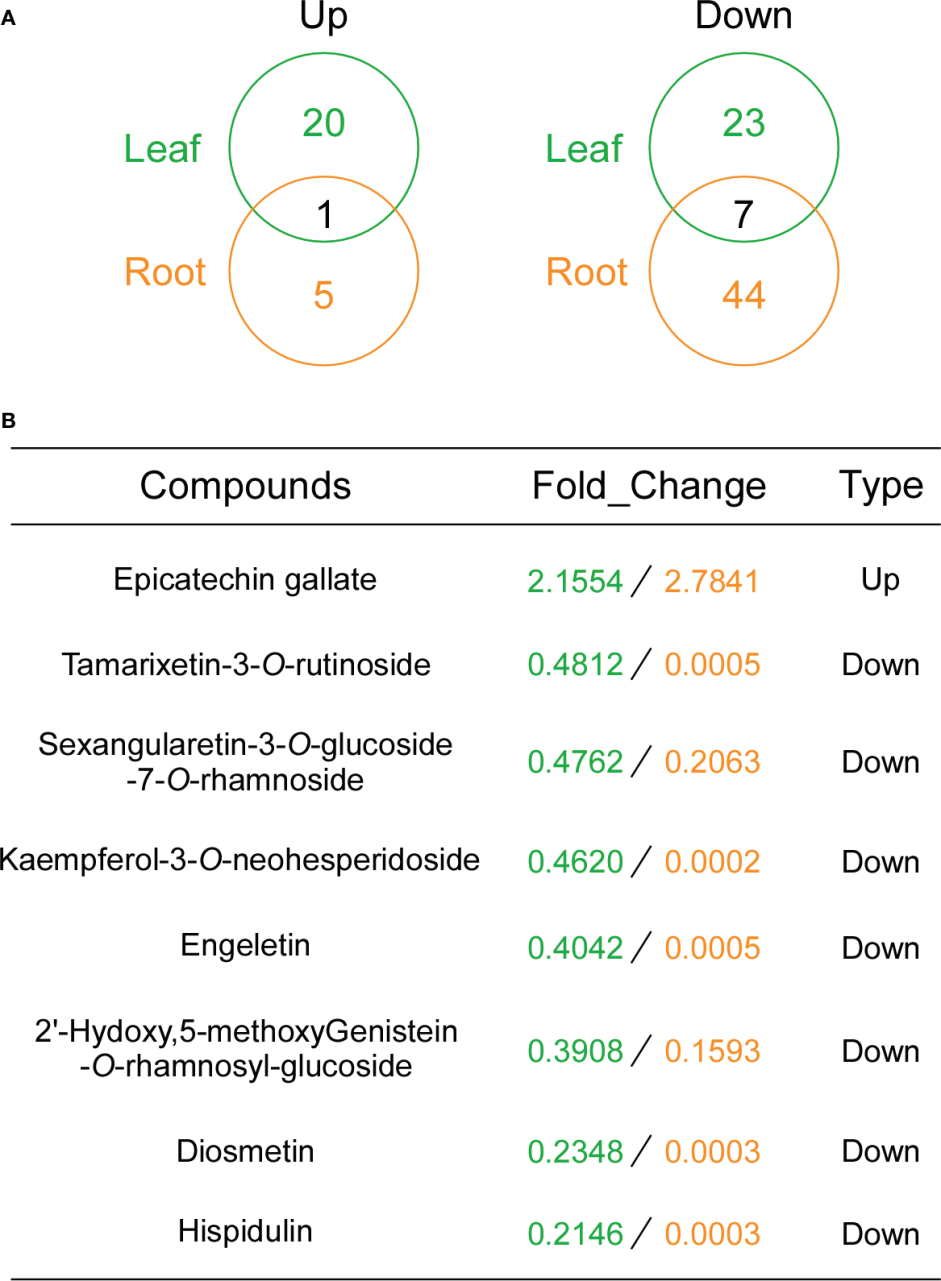
Flavonoid contents in transgenic tobacco leaves and roots. **(A)** Venn diagram analysis of the upregulated (left) and downregulated (right) flavonoids in leaves and roots. The numbers of the differentially regulated flavonoids identified in leaves and roots of transgenic tobacco plants are shown in the segments of the Venn diagram. **(B)** Information regarding flavonoid compounds with the same change tendencies. Fold changes in leaves and roots are marked in green and yellow, respectively.

To determine whether overexpression of *GbDFR6* influences other flavonoid compounds other than anthocyanins, flavonoid contents in leaves and roots of transgenic and wild-type tobacco plants were evaluated. A total of 21 flavonoid compounds in transgenic tobacco leaves were upregulated, whereas 30 flavonoid compounds were downregulated when compared to wild-type tobacco leaves (**Figure 6A** and **Table S2**). Furthermore, 6 flavonoids were upregulated, whereas 51 flavonoids were downregulated in transgenic tobacco roots when compared to wild-type tobacco roots (**Figure 6A** and **Table S3**). Specifically, epicatechin gallate was upregulated in both transgenic tobacco leaves and roots. In contrast, seven flavonoids (Tamarixetin-3-*O*-rutinoside; Sexangularetin-3-*O*-glucoside-7-*O*-rhamnoside; Kaempferol-3-*O*-neohesperidoside; Engeletin; 2’-Hydoxy,5-methoxyGenistein-*O*-rhamnosyl-glucoside; Diosmetin; Hispidulin) were downregulated in both transgenic tobacco leaves and roots (**Figure 6B**). The common flavonoids identified in the leaves and roots may explain the auxin-related developmental defects observed in transgenic tobacco leaves and roots.

## Discussion

Most of the plant mutants defective in auxin transport or signaling exhibit phenotypes, including small plant sizes, long primary roots, and decreased lateral root numbers (Casimiro et al., 2001; Ni et al., 2011; Lavenus et al., 2013). In the present study, transgenic tobacco seedlings exhibited relatively low fresh weights. The lengths of the primary roots of transgenic tobacco seedlings were longer than those of the wild-type tobacco seedlings, and lateral roots in transgenic tobacco seedlings were fewer than those in the wild-type tobacco seedlings. Notably, exogenous auxin treatment mitigated (or partially mitigated) the lateral root defects in transgenic tobacco seedlings. Therefore, transgenic tobacco plants overexpressing *GbDFR6* may have defects in auxin transport or signaling.

Intercellular transport of auxins is rapidly redirected following gravistimulation, in turn, resulting in the bending of plant roots (Tanimoto, 2005). Plants defective in polar auxin transport often exhibit varying degrees of loss of gravitropic responses (Rashotte et al., 2000). Flavonoids have been reported to be involved in the regulation of root gravitropic responses by redirecting PIN-mediated polar auxin transport (Santelia et al., 2008). Considering the moderate defects in gravitropic responses of transgenic tobacco roots and the key role of DFR in the flavonoid biosynthetic pathway, we speculate that a flavonoid (or an anthocyanin) accumulates in the transgenic tobacco plants and inhibits polar auxin transport in the roots, which ultimately results in the weaken of the gravitropic response phenotype.

Transgenic tobacco plants exhibited higher percentages of wrinkled leaves, especially medium and small leaves, when compared to wild-type tobacco plants. The finding indicates that a regulator of leaf flattening in transgenic tobacco plants was downregulated. The flattening of leaves to form broad blades is a key adaptation in plants and the phytohormone auxin plays a crucial role in the process (Guan et al., 2017). Therefore, our results demonstrated that a regulator associated with auxins was downregulated in transgenic tobacco plants.

Considering the occurrence of auxin-related defects in roots and leaves, one anthocyanin or flavonoid compound associated with consistent changes in roots and leaves could be the ideal candidate involved in auxin-related phenotypic changes in transgenic tobacco plants. Therefore, anthocyanin and flavonoid contents in the leaves and roots of transgenic and wild-type tobacco plants were determined. However, due to the extremely low anthocyanin content in roots (data not shown), only leaf anthocyanin content was determined. The results could indicate that the variations in anthocyanin contents may not explain the simultaneous appearance of auxin-related defects in transgenic tobacco roots and leaves. Notably, one flavonoid compound was upregulated, whereas seven flavonoid compounds were downregulated in both transgenic tobacco leaves and roots. Flavonoids have been associated with the regulation of auxin transport (Santelia et al., 2008). However, up to date, only one flavonoid compound (kaempferol 3-*O*-rhamnoside-7-*O*-rhamnoside) has been demonstrated to act as an endogenous polar auxin transport inhibitor in *Arabidopsis* (Yin et al., 2014). Consequently, more flavonoids could be involved in the regulation of auxin transport in plants, and the eight flavonoids identified in the present study are ideal candidates as novel regulators of auxin transport. Despite of auxin-related phenotypic changes between wild-type and transgenic tobacco plants, no significant differences in auxin contents were observed either in roots or in leaves. Thus, the candidate flavonoid may not change the overall auxin content in leaves or roots but disturb the local distribution of auxin.

In addition to auxin-related defects, transgenic tobacco plants exhibited the delayed flowering phenotype under SD conditions. *NtFT4* is considered the sole floral inducer in tobacco plants (Harig et al., 2012). The decreased expression levels of *NtFT4* in transgenic tobacco plants suggest that flowering time could be delayed through the FT pathway. Both wild-type and transgenic tobacco plants exhibited a similar flowering time under LD conditions, while the expression levels of *NtFT4* were lower in transgenic tobacco plants than in the wild-type tobacco plants, although the overall expression levels were low. A previous study revealed that the expression of *FT*-like genes is photoperiod-dependent even in the day-neutral tobacco varieties. Furthermore, the FT-like proteins regulate flowering under SD conditions; however, the molecular mechanisms underlying floral induction under LD conditions remain undetermined (Harig et al., 2012). Overall, the overexpression of *GbDFR6* in transgenic tobacco plants inhibits the expression of *NtFT4* under SD and LD conditions. The relatively low expression levels of *NtFT4* under SD conditions delayed the flowering time in transgenic tobacco plants through the FT pathway. However, floral induction is FT-independent under LD conditions, and the low expression level of *NtFT4* did not have an effect on flowering time in transgenic tobacco plants.

Flowering time in angiosperms is influenced by numerous environmental and endogenous factors, which are integrated and converge on central floral regulatory hubs (Kinoshita and Richter, 2020). The FT and TFL1 homologs in the FT/TFL1 family, which functions in an inverse manner, influences the signals to regulate flowering time (Wickland and Hanzawa, 2015). Recent studies have demonstrated that gymnosperms also have *FT*-like and *TFL1*-like genes (Liu et al., 2016). Furthermore, several key genes involved in the FT pathway have been isolated and characterized in ginkgo, a perennial gymnosperm (Yan et al., 2017; Wang et al., 2019). Considering that the overexpression of *GbDFR6* delayed flowering time in transgenic tobacco plants by inhibiting the expression of *NtFT4*, *GbDFR6* could be a key factor influencing the regulation of flowering time in ginkgo by altering anthocyanin (or flavonoid) content, which ultimately alters the expression levels of *GbFT* genes. The regulation of flowering time by anthocyanins (or flavonoids) has been reported in previous studies. Transgenic tobacco plants overexpressing *CsDFR* and *CsANR* (anthocyanidin reductase) exhibited high flavonoid contents and early flowering times (Kumar et al., 2013). Thus, we hypothesize that anthocyanins (or flavonoids) regulate *FT* gene expression in an inverse manner in some plant species. Similar to FT and TFL1 proteins, one anthocyanin (or flavonoid) promotes flowering, whereas the other represses flowering. In wild-type and most transgenic plants, the two forces offset each other and therefore, flowering time is not easily affected by alteration of anthocyanin (or flavonoid) levels. However, the overexpression of specific genes (*GbDFR6*, *CsDFR*, and *CsANR*) may disrupt the balance and adjust the flowering time in transgenic plants.

Transgenic tobacco plants overexpressing *GbDFR6* exhibit unrelated auxin and flowering phenotypes simultaneously. DFR catalyzes the first committed reaction leading to anthocyanin production, and it is considered a key enzyme in the regulation of the carbon flux direction in anthocyanin production (Saito et al., 2013). Therefore, the product of *GbDFR6* could be a multifunctional molecule involved in the regulation of auxin transport (signaling) and flowering. Alternatively, because the flavonoid biosynthetic pathway is a complex network with many interrelated steps, the overexpression of *GbDFR6* may also influence the expression levels of other flavonoid biosynthesis-related genes, which causes the variations in the contents of other flavonoids and ultimately leads to multiple phenotypical variations in transgenic plants. Consistent with this hypothesis, the contents of several flavonoids, which are not direct products of DFR, were altered in transgenic plants. Similarly, the overexpression of *GbF3’H1* in transgenic poplar (*Populus davidiana*) alters the expression of other flavonoid biosynthesis-related genes (Wu et al., 2020).

## Conclusion

In this research, the phenotypic changes in transgenic tobacco plants overexpressing *GbDFR6* were investigated. Pleiotropic defects in root and leaf development indicated impaired auxin transport in transgenic tobacco plants. Delayed flowering in transgenic tobacco plants signified the involvement of anthocyanins (or flavonoids) in the regulation of flowering time. UPLC-ESI-MS/MS analysis provided novel candidate for regulating auxin transport and flowering time. These results indicated novel and multiple roles of *GbDFR6* in ginkgo.

As an important economic crop, flowering time in tobacco plants has a substantial impact on its biomass production. According to the results of the present study, the overexpression of *GbDFR6* delayed flowering time in transgenic tobacco plants. Nevertheless, the molecular mechanisms underlying the phenomenon remain unclear, and require further studies. Our results provide a valuable method in tobacco industry.

## Data availability statement

The original contributions presented in the study are included in the article/**Supplementary Material**. Further inquiries can be directed to the corresponding author.

## Author contributions

JN and MX conceived and designed the experiments. NZ, YZ, KD, PQ, XW and WD performed the experiments. JN, NZ and MX analyzed the data. YZ contributed reagents/materials/analysis tools. JN and MX wrote the manuscript. All authors contributed to the article and approved the submitted version.

## Supplementary material

Supplementary Figure 1 The induction of lateral root of transgenic tobacco seedlings by exogenous auxin treatment. **(A)** Untreated control. **(B)** NAA treatment (10^-7^ M). Bars = 2 cm.

Supplementary Figure 2 Comparison of internal auxin contents in leaves and roots (mean ± SD; n = 6; NS, not significant, *P* > 0.05; Student’s *t*-test).

Supplementary Figure 3 A comparative analysis of flowering time between wild-type and transgenic tobacco plants under long-day conditions. **(A)** Similar flowering times between wild-type and transgenic tobacco plants. **(B)** A comparison of flowering days between wild-type and two transgenic tobacco lines (NS, not significant, *P* > 0.05; Student’s *t*-test). **(C-D)** Expression of *NtFT2* **(C)** and *NtFT4* **(D)** in tobacco plants before bolting as determined by quantitative reverse transcription polymerase chain reaction (data are presented as means ± SD; n = 4; * indicates significant differences, *P* < 0.01; Student’s *t*-test).

Supplementary Figure 4 A comparison of cyanidin-3,5-O-diglucoside contents between wild-type and transgenic tobacco leaves with three biological repetitions.

Supplementary Table 1 Primers used for quantitative reverse transcription polymerase chain reaction.

Supplementary Table 2 Upregulated and downregulated flavonoid compounds detected in tobacco leaves. Common flavonoids detected in roots are marked in bold.

Supplementary Table 3 Upregulated and downregulated flavonoid compounds detected in tobacco roots. Common flavonoids detected in leaves are marked in bold.
