# Supplementary figures and images for: Transgenic tobacco plant overexpressing ginkgo dihydroflavonol 4-reductase gene GbDFR6 exhibits multiple developmental defects
Source: Front Plant Sci. 2022 Dec 14;13:1066736. doi: 10.3389/fpls.2022.1066736 (PMC9794611; doi:10.3389/fpls.2022.1066736)

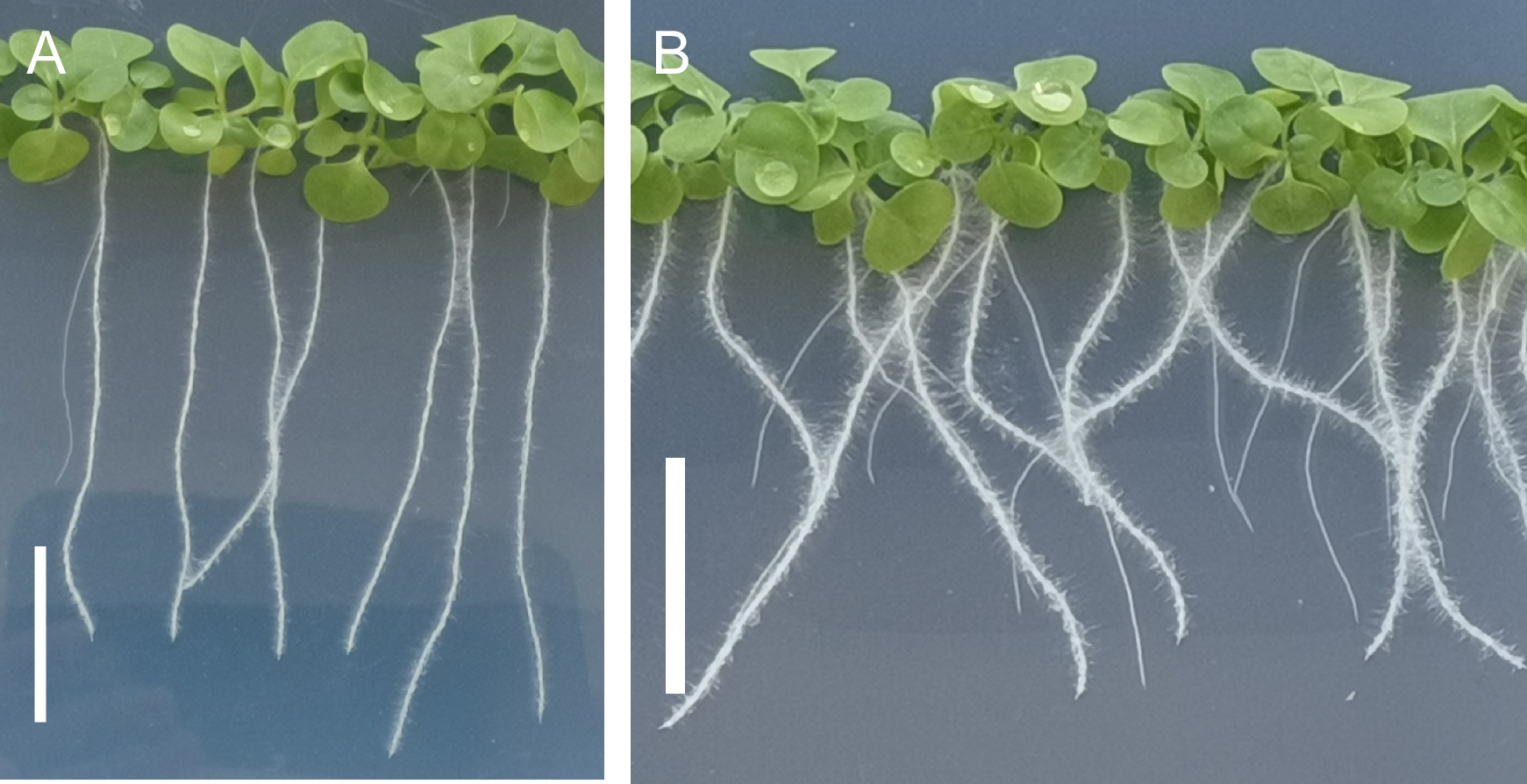

Supplement: Supplementary Figure 1 — The induction of lateral root of transgenic tobacco seedlings by exogenous auxin treatment. (A) Untreated control. (B) NAA treatment (10-7 M). Bars = 2 cm. [file Image_1.tif]

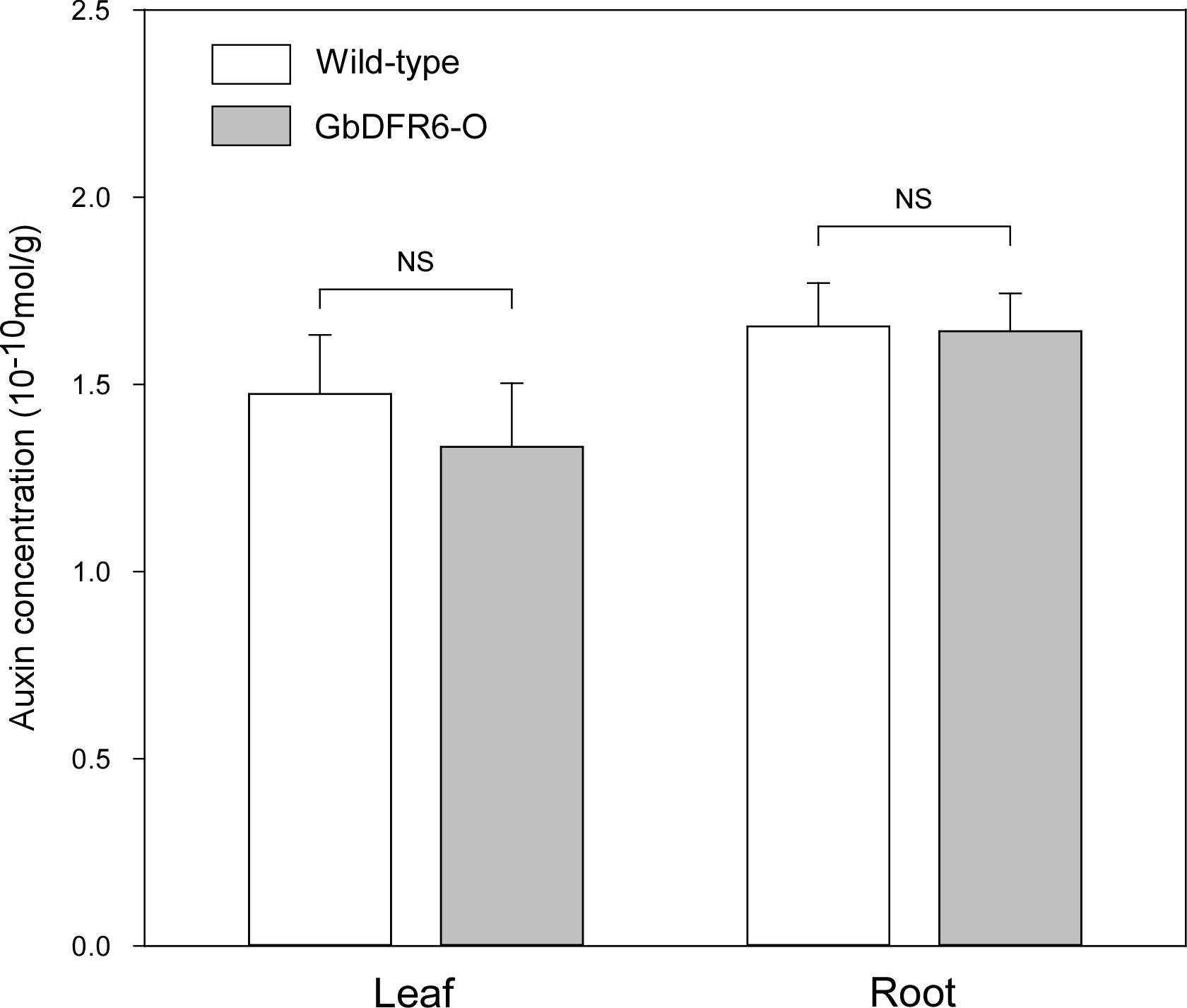

Supplement: Supplementary Figure 2 — Comparison of internal auxin contents in leaves and roots (mean ± SD; n = 6; NS, not significant, P > 0.05; Student’s t-test). [file Image_2.tif]

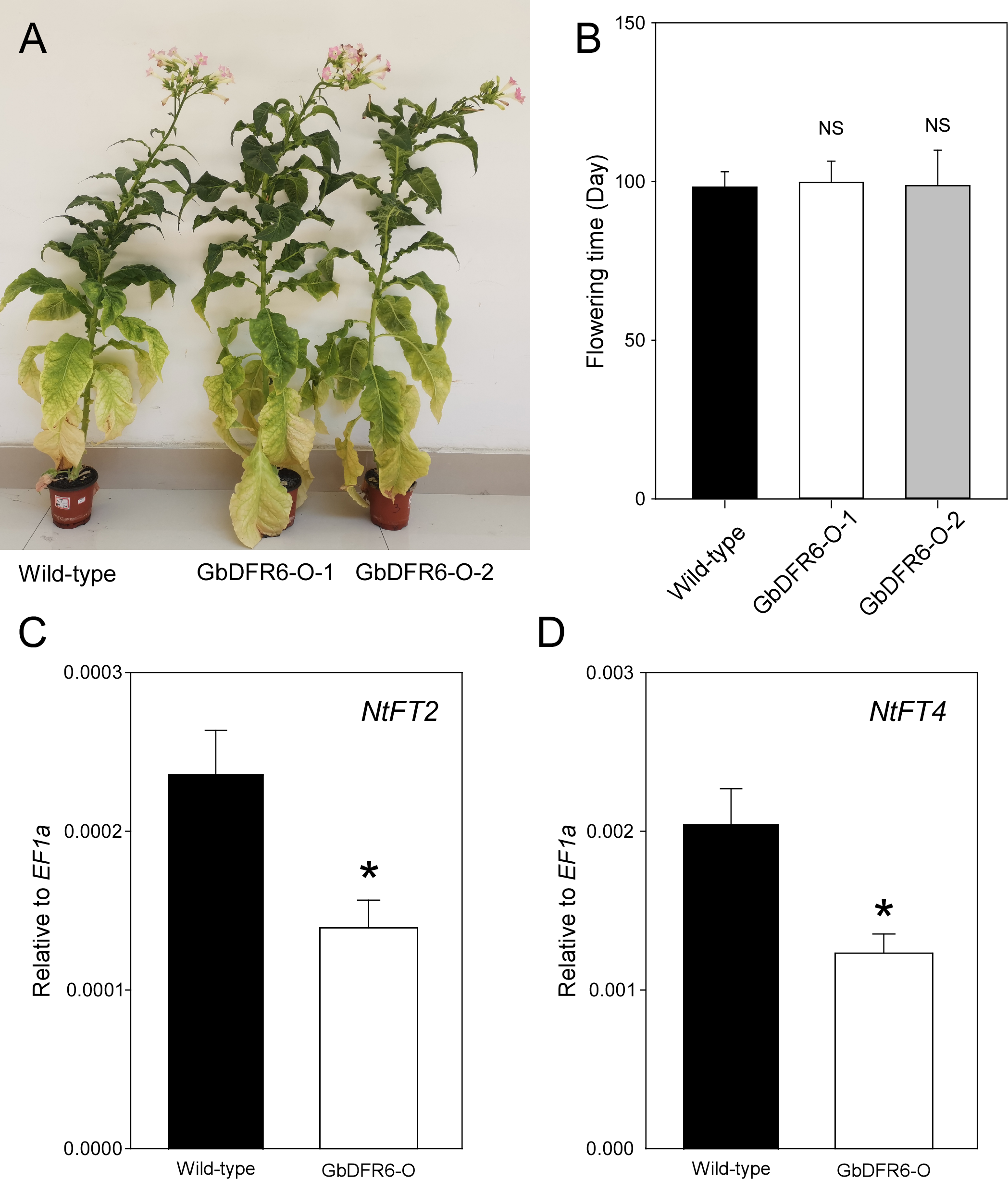

Supplement: Supplementary Figure 3 — A comparative analysis of flowering time between wild-type and transgenic tobacco plants under long-day conditions. (A) Similar flowering times between wild-type and transgenic tobacco plants. (B) A comparison of flowering days between wild-type and two transgenic tobacco lines (NS, not significant, P > 0.05; Student’s t-test). (C-D) Expression of NtFT2 (C) and NtFT4 (D) in tobacco plants before bolting as determined by quantitative reverse transcription polymerase chain reaction (data are presented as means ± SD; n = 4; * indicates significant differences, P < 0.01; Student’s t-test). [file Image_3.tif]

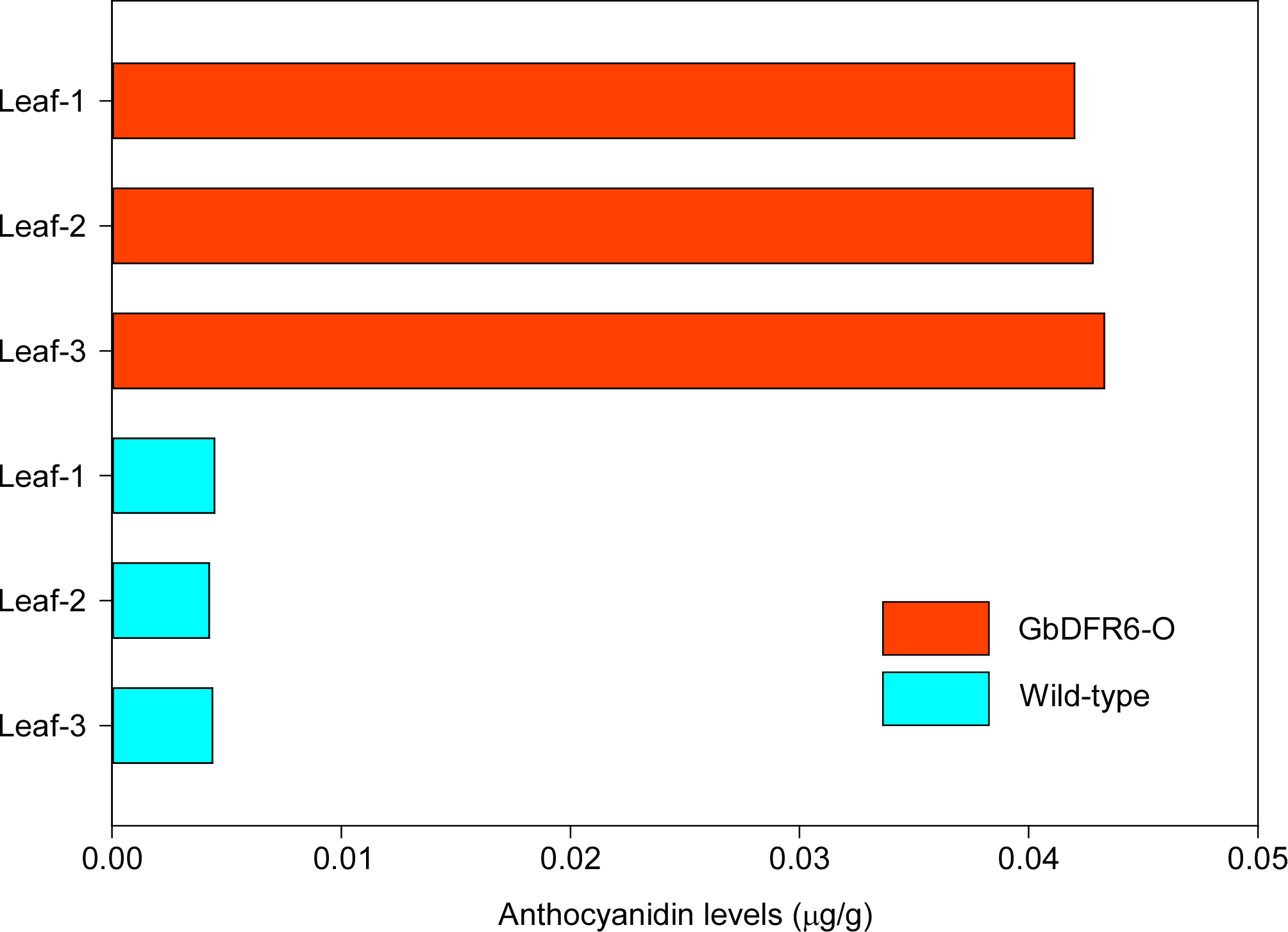

Supplement: Supplementary Figure 4 — A comparison of cyanidin-3,5-O-diglucoside contents between wild-type and transgenic tobacco leaves with three biological repetitions. [file Image_4.tif]
